# Intermediate-term follow-up of laparoscopic pectopexy cases and their effects on sexual function and quality of life: a cross-sectional study

**DOI:** 10.1590/1516-3180.2021.0488.R1.171121

**Published:** 2022-06-06

**Authors:** Selami Erdem

**Affiliations:** I MD. Physician, Department of Gynecology and Obstetrics, Özel Bağlar Hastanesi, Diyarbakır, Turkey

**Keywords:** Pelvic organ prolapse, Quality of life, Laparoscopy, Pectopexy, Prolapsus, Laparoscopic surgery

## Abstract

**BACKGROUND:**

Apical prolapsus refers to downward displacement of the vaginal apex, uterus or cervix. Pelvic organ prolapse (POP) can significantly affect women’s daily activities and sexuality.

**OBJECTIVE:**

To investigate, at the mid-term follow-up after laparoscopic pectopexy surgery, whether this procedure improved the patients’ quality of life and sexual function.

**DESIGN AND SETTING:**

In this cross-sectional study, data on patients who underwent laparoscopic pectopexy in the Gazi Yasargil Education and Research Hospital were evaluated.

**METHODS:**

Thirty-five patients with symptomatic apical prolapse and POP quantification stage II and higher were included in this study. We used the Turkish version of the female sexual function index (FSFI) questionnaire to assess preoperative and postoperative sexual dysfunction, and the Turkish version of the Prolapse Quality of Life Questionnaire (P-QOL) to evaluate the severity of POP and its impact on quality of life.

**RESULTS:**

The mean age, parity and length of follow-up of the patients were 36.08 ± 9.04 years, 4.00 ± 1.86 and 28.88 ± 5.88 months, respectively. The most common complications were de novo rectocele in three patients (8.6%) and de novo cystocele in two patients (5.7%). All the FSFI and P-QOL scores were statistically significantly improved in the postoperative period (P < 0.001 for all scores of both FSFI and P-QOL).

**CONCLUSION:**

The quality of life and sexual function of the patients who underwent laparoscopic pectopexy were found to have become statistically improved at the midterm follow-up. Laparoscopic pectopexy was found to be a viable, effective and safe procedure.

## INTRODUCTION

Apical prolapse refers to downward displacement of the vaginal apex, uterus or cervix. Pelvic organ prolapse (POP) affects 50% of parous women, and this rate increases with age, menopause and parity. However, POP can be asymptomatic and may only be noticed when patients are examined for another reason.^1^ POP can significantly affect women’s daily activities and sexuality. Many studies have reported that sacropexy is the most appropriate approach for providing a physiological axis for the vagina in terms of size, depth and inclination.^2,3,4^ However, defecation disorders and urinary problems are common after sacropexy.

The pectopexy procedure, defined as a new endoscopic prolapse surgery method, was developed especially for obese patients by Banerjee and Noe in 2007. In this, mesh fixation is performed on both sides of the descending lateral parts of the iliopectineal ligament, for suspension of the cervix or vagina. This segment of the ligament is located at the level of the second sacral vertebra (S2), which is the most suitable level for the physiological axis of the vagina. In this method, because the mesh does not cross the ureter or intestine and passes through the broad ligament, it does not cause problems with the ureter and intestine. In addition, the hypogastric vessels are at a safe distance from any danger. This new method is a simpler and safer procedure, especially in patients for whom surgery is difficult.^5^

## OBJECTIVE

In this study, our aim was to investigate, at the mid-term follow-up after laparoscopic pectopexy surgery, whether this procedure improved the patients’ quality of life and sexual function; and to determine the reliability, applicability and effectiveness of the surgery by using the female sexual function index (FSFI) and Prolapse Quality of Life (P-QOL) questionnaires.

## METHODS

### Study design and patients

Data on patients who underwent laparoscopic pectopexy in our hospital between January 2016 and June 2018 were collected from the registry system of our hospital. Approval was obtained from the Ethics Committee of Gazi Yasargil Education and Research Hospital (decision no. 507; date: March 7, 2020). In this study, which we conducted in accordance with the Declaration of Helsinki, we obtained written informed consent from all participants. All the surgical operations were performed by three gynecological surgeons with advanced laparoscopic experience.

The patients with apical prolapse were evaluated in terms of their feeling of pressure in the vagina, bloated/bulging sensation, urinary symptoms, constipation and sexual dysfunction, and the results were recorded. Genital prolapse was evaluated using both physical examination and ultrasonography.

The pelvic organ prolapse quantification system (POP-Q) was used for prolapse evaluation. Only patients with symptomatic prolapse (POP-Q stage II and higher) were included in this study. Patients with pelvic inflammatory disease, genital malignancy, pregnancy or previous POP surgery were excluded from the study.

The Turkish version of the FSFI questionnaire, evaluating six sexual desire domains (sexual desire, sexual arousal, lubrication, orgasm, satisfaction and pain), was used to evaluate preoperative and postoperative sexual dysfunction. In this questionnaire, the lowest score is 2, and the highest score is 36. Total scores < 26.55 were considered indicative of impaired sexual function.^6,7^

The Turkish version of the P-QOL questionnaire, which is a reliable, consistent and valid tool, was used to evaluate the severity of POP and its effect on quality of life. A high P-QOL score represents poor quality of life.

### Surgical procedure

All the operations were performed under endotracheal general anesthesia in the dorsal lithotomy position. After the operation had been started and trocars had been placed, the patient was placed in the Trendelenburg position. Cephazolin sodium (1 g) was administered to all the patients preoperatively, and a Foley catheter was placed in the bladder.

**Figure 1.**
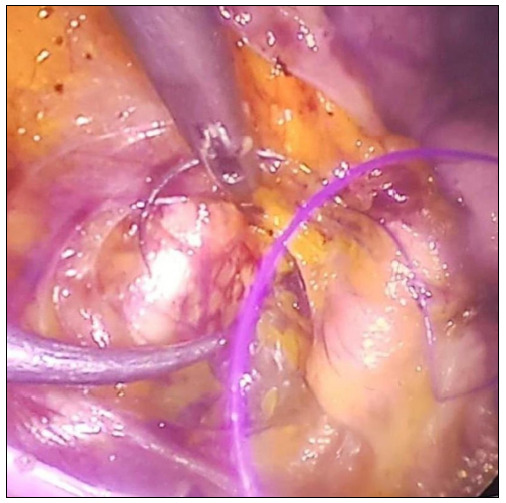
Iliopectineal ligament fixation.

Firstly, a camera was inserted through a 10-mm periumbilical trocar. Pneumoperitoneum was created until an intra-abdominal pressure of 13 mmHg was achieved. Then, two 5-mm trocars were placed ipsilaterally on the left side of the patient; and one 5-mm trocar, on the right side. During the operation, the surgeon stood on the left side of the patient, and the assistant stood on the right side. A uterine manipulator was used in all the patients, to position the uterus. Monopolar cautery was used for the dissection. The bladder was dissected starting from the uterus, using sharp and blunt dissection. On both sides, the lateral part of the iliopectineal ligament was reached, up to the area bounded by the ligamentum rotundum, external iliac vein and obturator nerve (Figure 1).

**Figure 2.**
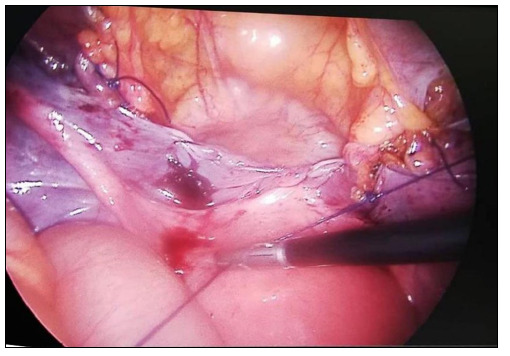
Closing the peritoneal layer.

The polypropylene mesh that was brought into the abdomen from a 10-in trocar was first fixed with a non-absorbable polypropylene monofilament suture on the lateral part of both iliopectineal ligaments, in a tension-free manner. Then, the mesh was fixed to the lower anterior segment of the uterus with three non-absorbable polypropylene monofilament sutures. The operation was completed by closing the peritoneal layer with no. 0 absorbable sutures (Figure 2).

### Statistical analysis

We performed all the statistical analyses using the SPSS software (version 26.0; SPSS Inc., Chicago, Illinois, United States). Demographic data were calculated using descriptive statistics. Means and standard deviations were used to describe the data. The Kolmogorov-Smirnov test was used to verify whether an assumption of normal distribution of variables could be made. Paired t tests were used to compare P-QOL and FSFI scores before and after the pectopexy.

## RESULTS

The mean age, parity and length of follow-up of the patients included in the study were 36.08 ± 9.04 years, 4.00 ± 1.86 and 28.88 ± 5.88 months, respectively (Table 1). The duration of surgery (mean ± standard deviation, SD) was 71.34 ± 18.33 minutes, while the mean blood loss was 94.00 ± 74.36 ml. Except for three patients, all the cases were stages 2 and 3, and the most common additional procedure was anterior colporrhaphy, which was performed in 45.7% of all the cases (Table 2).

**Table 1.** Demographic and clinical characteristics of the patients

Characteristics	(Mean ± standard deviation)
Age (years)	36.08 ± 9.04
Parity	4.00 ± 1.86
Length of follow-up (months)	28.88 ± 5.88

**Table 2.** Clinical characteristics of the subjects included in the study

Characteristics	
**Duration of surgery (min), mean ± SD**	71.34 ± 18.33
**Blood loss (ml), mean ± SD**	94.00 ± 74.36
**Preoperative POP-Q, n (%)**	
Stage 2	16 (45.7)
Stage 3	16 (45.7)
Stage 4	3 (8.60)
**Additional procedures, n (%)**	
Anterior colporrhaphy	16 (45.7)
Posterior colporrhaphy	9 (25.7)
Sling operation	7 (20.0)
Tubal ligation	3 (8.6)

POP-Q = pelvic organ prolapse quantification system; min = minute; ml = milliliter; SD = standard deviation.

In the postoperative period, de novo rectocele was found in three patients (8.6%); and de novo cystocele, in two patients (5.7%). Only one patient (2.9%) had complications, namely urinary infection, de novo stress urinary incontinence, relapse, de novo urgency and de novo constipation (Table 3).

**Table 3.** Complications observed in patients in the postoperative period

Characteristics	n (%)
Urinary infection	1 (2.9)
De novo stress urinary incontinence	1 (2.9)
Relapse	1 (2.9)
De novo urgency	1 (2.9)
De novo cystocele	2 (5.7)
De novo rectocele	3 (8.6)
De novo constipation	1 (2.9)

All the FSFI and P-QOL scores were found to have become statistically significantly improved in the postoperative period (P < 0.001 for all scores of both FSFI and P-QOL). In addition, the total FSFI score was 28.47 ± 2.40 in the postoperative period, which exceeded the cutoff score of 26.5 (Tables 4 and 5).

**Table 4.** Female sexual function index (FSFI) scores

Characteristics	Preoperative (Mean ± SD)	Postoperative (Mean ± SD)	Significant (P-value)
Desire	2.69 ± 0.87	4.83 ± 0.62	< 0.001
Arousal	2.82 ± 1.12	4.90 ± 0.63	< 0.001
Lubrication	2.70 ± 0.95	5.00 ± 0.40	< 0.001
Orgasm	2.44 ± 0.93	4.52 ± 0.43	< 0.001
Satisfaction	3.02 ± 0.64	4.54 ± 0.69	< 0.001
Pain	3.47 ± 0.50	4.66 ± 0.69	< 0.001
Total score	16.95 ± 4.45	28.47 ± 2.40	< 0.001

SD = standard deviation.

**Table 5.** Prolapse Quality of Life (P-QOL) scores observed in the patients included in the study

Characteristics	Preoperative Mean ± SD	Postoperative Mean ± SD	Significant P-value
GHP	5.94 ± 0.58	3.35 ± 0.37	< 0.001
PI	23.68 ± 4.65	8.00 ± 2.75	< 0.001
RL	3.16 ± 0.86	1.82 ± 1.42	< 0.001
PL	4.50 ± 1.45	1.80 ± 0.86	< 0.001
SL	3.51 ± 0.71	1.39 ± 0.70	< 0.001
PR	6.20 ± 1.78	1.79 ± 1.18	< 0.001
EM	6.25 ± 1.47	2.90 ± 0.96	< 0.001
SE	2.70 ± 0.43	1.50 ± 0.80	< 0.001
SM	6.43 ± 1.14	2.04 ± 0.93	< 0.001
GS	62.41 ± 12.25	24.62 ± 7.04	< 0.001

SD = standard deviation; GHP = general health perceptions; PI = prolapse impact; RL = role limitations; PL = physical limitations; SL = social limitations; PR = personal relationships; EM = emotions; SE = sleep/energy; SM = severity measurements; GS = general score.

## DISCUSSION

In this study, we investigated at the mid-term follow-up after laparoscopic pectopexy surgery whether this procedure improved the patients’ quality of life and sexual function; and determined the reliability, applicability and effectiveness of the surgery. We detected that all the FSFI and P-QOL scores of the patients included in the study became statistically significantly improved in the postoperative period.

Laparoscopic pectopexy is a new type of endoscopic prolapse surgery. Both abdominal and laparoscopic sacrocolpopexy for apical prolapse surgery have been reported to be associated with excellent anatomical and functional outcomes over the long term.^8,9,10^ However, potential problems may be observed, including pelvic outlet stenosis, hypogastric nerve damage, sigmoid colon damage, ureter damage, osteomyelitis and sacrohysteropexy. As the lateral parts of the iliopectineal ligament are used in mesh fixation in pectopexy, fewer long-term problems are expected.^11^ In addition, pelvic outlet stenosis, ureteral and hypogastric nerve damage and de novo constipation are not expected with this method.

In a previous study, de novo constipation was detected in the pectopexy group, while constipation was found in 19.5% of the patients in the sacropexy group. In that study, no statistically significant difference was found between the pectopexy and sacropexy groups in terms of the incidence of de novo rectocele (9.5% versus 9.8%, respectively).^12^ Similarly to the reason for the low de novo constipation rate in our study, the possible reason for the absence of de novo constipation in that study may have been the absence of pelvic outlet stenosis and hypogastric nerve damage. Pregnancy does not affect the success of pectopexy, which is thus a safe method for women who desire fertility.^13^

Pectopexy may be protective against de novo anterior and lateral defects owing to the lateral location of the mesh.^12^ Similarly, we found a low incidence rate for de novo cystocele in our study (5.7%) and did not observe any de novo lateral defects. Although many studies have reported high incidence rates (> 25%) of de novo stress urinary incontinence (SUI) after sacrocolpopexy, we found that the incidence rate of de novo SUI was low (2.9%) in our study.^14,15,16^ It has been have reported that pectopexy is not associated with increased intraoperative risk.^11^ Similarly, in our study, no intraoperative complications developed, except in one patient who underwent laparotomy due to intraoperative bleeding.

Similar to the low recurrence rates reported in cases of laparoscopic sacrocolpopexy, recurrence was only found in one patient at our midterm follow-up.^14,17^ Use of a mesh in surgical treatments for cystocele and POP may cause dyspareunia and worsen sexual function.^18,19^ It has been suggested in some studies that patients’ quality of life and sexual function rarely improve after POP surgery.^18,20^ However, we detected that the quality of life and sexual function of our patients improved.

Unlike in a previous study in which the mesh was fixed to both iliopectineal ligaments with two stitches, we provided fixation to each ligament with one suture. We observed that this could be an effective, safe and applicable method.^5^

The limitations of our study were that it was conducted at a single center and that the number of cases was small. On the other hand, the strength of our study was that laparoscopic pectopexy was presented as an easily applicable, effective and safe procedure that at the midterm follow-up can be seen to have improved the quality of life and sexual function of patients with POP.

## CONCLUSION

In this study, we detected at the midterm follow-up that the quality of life and sexual function of patients who underwent laparoscopic pectopexy were statistically improved. In addition, laparoscopic pectopexy was found to be a viable, effective and safe procedure. Therefore, it can be considered to be an alternative treatment method for sacropexy in suitable patients with POP.
